# Supplementary material for: Drug Repurposing Using Gene Co-Expression and Module Preservation Analysis in Acute Respiratory Distress Syndrome (ARDS), Systemic Inflammatory Response Syndrome (SIRS), Sepsis, and COVID-19
Source: Biology (Basel). 2022 Dec 15;11(12):1827. doi: 10.3390/biology11121827 (PMC9775208; doi:10.3390/biology11121827)
Supplement: Supplementary file 1 [file biology-11-01827-s001.zip › biology-2038639-supplementary.pdf]

Supplementary Information

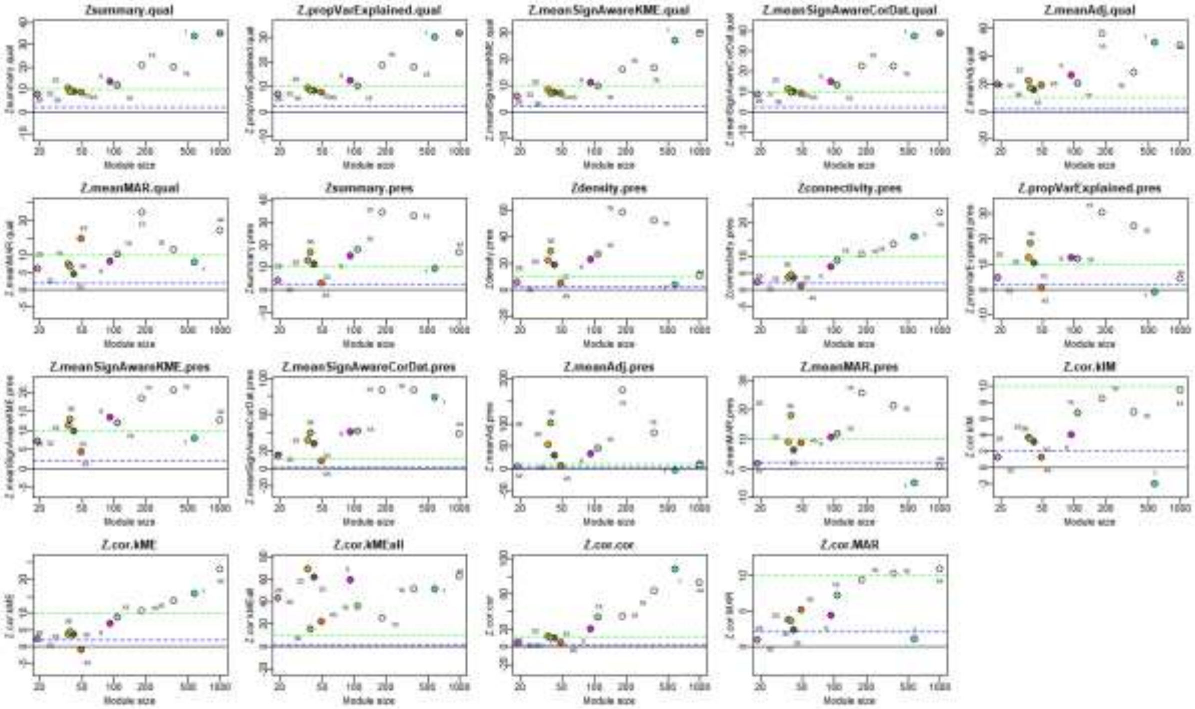

Figure S1. Summary of module quality statistics (first row; names ending with suffix .qual) and module preservation (second and third row) of sepsis modules. Modules are labeled by color and numeric value (Brown – 46; dark olive green – 33; dark orange – 45; floral white – 44; light cyan – 16; light green – 18; light yellow – 19; magenta – 9; orange – 25; sky blue – 28; turquoise – 1; violet – 32; yellow green – 36).

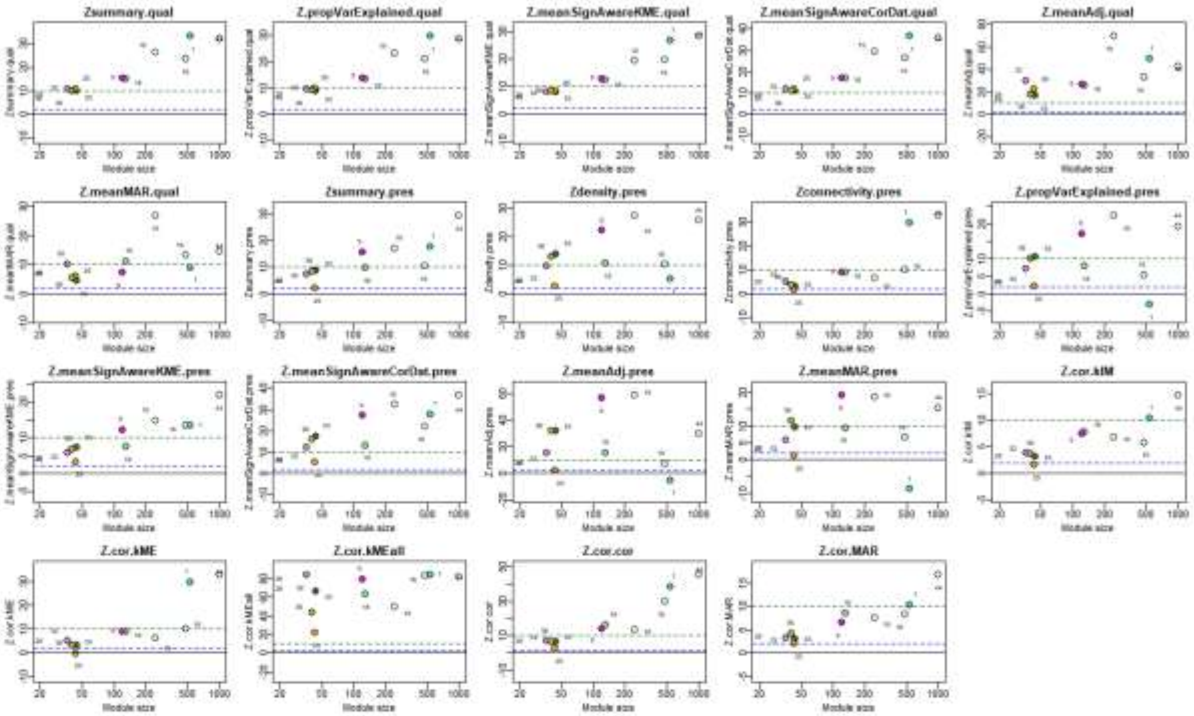

Figure S2. Summary of module quality statistics (first row; names ending with suffix .qual) and module preservation (second and third row) of SIRS modules. Modules are labeled by color and numeric value (Brown – 46; dark olive green – 33; dark orange – 45; floral white – 44; light cyan – 16; light green – 18; light yellow – 19; magenta – 9; orange – 25; sky blue – 28; turquoise – 1; violet – 32; yellow green – 36).

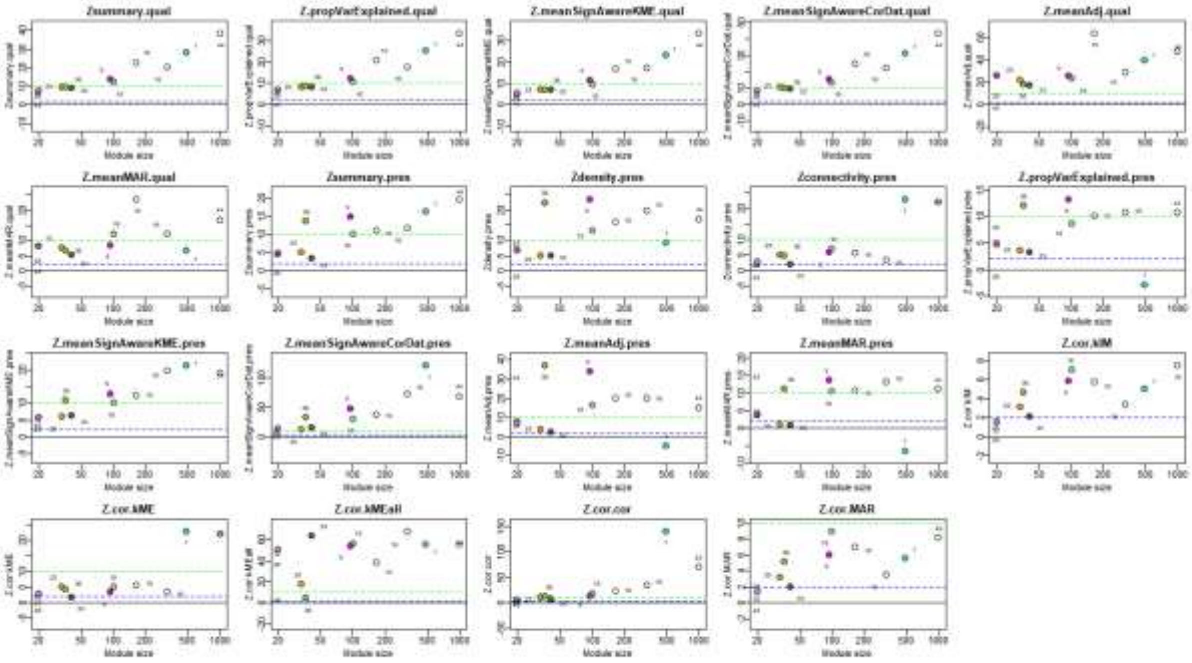

Figure S3. Summary of module quality statistics (first row; names ending with suffix .qual) and module preservation (second and third row) of COVID-19 modules. Modules are labeled by color and numeric value (Brown – 46; dark olive green – 33; dark orange – 45; floral white – 44; light cyan – 16; light green – 18; light yellow – 19; magenta – 9; orange – 25; sky blue – 28; turquoise – 1; violet – 32; yellow green – 36).

Table S1. Summary of functional GO biological process enrichment results for the thirteen identified modules.

Module	Color	Size	Top GO Biological Process (P ≤)	Hub Gene
1	Turquoise	2803	GO:0050911 Chemical Stimulus Detection (2.88E-18)	TRIM49D2
9	Magenta	410	GO:0002250 Adaptive Immune Response (2.69E-48)	TRAJ12
16	Light Cyan	915	GO:0050794 Modulation of Cellular Process (1.20E-20)	ACAP2
18	Light Green	265	GO:0044419 Response to Organism (2.85E-54)	STAT2
19	Light Yellow	455	GO:0009056 Catabolic Processes (4.82E-17)	SLC1A5
25	Orange	123	GO:0000278 Mitotic Cell Cycle (7.96E-63)	TOP2A
28	Sky Blue	100	GO:0002250 Adaptive Immune Response (5.11E-69)	IGKV1-6
32	Violet	94	GO:0042113 B-cell Activation (3.40E-12)	AFF3
33	Dark Olive Green	91	GO:0000902 Cell Morphogenesis (5.27E-3)	LRP1
36`	Yellow Green	87	GO:0030168 Platelet Activation (1.38E-11)	TREML1
44	Floral White	6699	GO:0044260 Cellular Macromolecule Metabolic Process (7.81E-16)	METTL23
45	Dark Orange	120	GO:0006396 RNA Processing (7.03E-80)	SNORD104
46	Brown	39	GO:0007283 Spermatogenesis (3.71E-12)	TSPY4

Note: Hub gene corresponds to the gene with the highest intra-module connectivity. Size refers to the number of genes in a module.

Table S2. Summary of functional GO molecular function enrichment results for the thirteen identified modules.

Module	Color	Size	Top GO Molecular Function (P ≤)	Hub Gene
1	Turquoise	2803	GO:0004984 Olfactory receptor activity (1.80E-17)	TRIM49D2
9	Magenta	410	GO:0042605 Peptide antigen binding (1.56E-09)	TRAJ12
16	Light Cyan	915	GO:0140096 Catalytic activity, acting on a protein (4.69E-14)	ACAP2
18	Light Green	265	GO:0003725 Double-stranded RNA binding (1.22E-07)	STAT2
19	Light Yellow	455	GO:0005515 Protein binding (2.15E-14)	SLC1A5

25	Orange	123	GO:0008017 Microtubule binding (4.76E-14)	TOP2A
28	Sky Blue	100	GO:0003823 Antigen binding (5.15E-63)	IGKV1-6
32	Violet	94	GO:0032395 MHC class II receptor activity (3.22E-06)	AFF3
33	Dark Olive Green	91	GO:0016502 Nucleotide receptor activity (4.88E-02)	LRP1
36	Yellow Green	87	GO:0032561 Guanyl ribonucleotide binding (7.62E-03)	TREML1
44	Floral White	6699	GO:0005515 Protein binding (1.35E-18)	METTL23
45	Dark Orange	120	GO:0030627 Pre-mRNA 5'-splice site binding (6.00E-03)	SNORD104
46	Brown	39	GO:0008494 Translation activator activity (1.17E-07)	TSPY4

Note: Hub gene corresponds to the gene with the highest intra-module connectivity. Size refers to the number of genes in a module.

Table S3. Summary of functional GO cellular component enrichment results for the thirteen identified modules.

Module	Color	Size	Top GO Cellular Component (P ≤)	Hub Gene
1	Turquoise	2803	GO:0016021 Integral component of membrane (4.87E-07)	TRIM49D2
9	Magenta	410	GO:0042101 T-cell receptor complex (2.02E-49)	TRAJ12
16	Light Cyan	915	GO:0005737 Cytoplasm (8.12E-35)	ACAP2
18	Light Green	265	GO:0005737 Cytoplasm (1.90E-20)	STAT2
19	Light Yellow	455	GO:0005737 Cytoplasm (1.21E-29)	SLC1A5
25	Orange	123	GO:0005694 Chromosome (2.07E-41)	TOP2A
28	Sky Blue	100	GO:0019814 Immunoglobulin complex (2.01E-99)	IGKV1-6
32	Violet	94	GO:0005887 Integral component of plasma membrane (3.91E-08)	AFF3
33	Dark Olive Green	91	GO:0031982 Vesicle (3.54E-07)	LRP1
36	Yellow Green	87	GO:0031091 Platelet alpha granule (1.52E-10)	TREML1
44	Floral White	6699	GO:0005654 Nucleoplasm (6.08E-22)	METTL23
45	Dark Orange	120	GO:0005730 Nucleolus (3.14E-10)	SNORD104

46	Brown	39	GO:0005634 Nucleus (1.59E-4)	TSPY4

Note: Hub gene corresponds to the gene with the highest intra-module connectivity. Size refers to the number of genes in a module.

Table S4. Summary of pathway enrichment results for the thirteen identified modules.

Module	Color	Size	Top KEGG Pathway (P ≤)	Hub Gene
1	Turquoise	2803	KEGG:04740 Olfactory transduction (1.32E-13)	TRIM49D2
9	Magenta	410	KEGG:04658 Th1 and Th2 cell differentiation (3.23E-14)	TRAJ12
16	Light Cyan	915	KEGG:04062 Chemokine signaling pathway (3.97E-06)	ACAP2
18	Light Green	265	KEGG:04621 NOD-like receptor signaling pathway (1.12E-10)	STAT2
19	Light Yellow	455	KEGG:04137 Mitophagy-animal (1.39E-06)	SLC1A5
25	Orange	123	KEGG:04110 Cell cycle (4.68E-14)	TOP2A
28	Sky Blue	100	No significant KEGG pathway.	IGKV1-6
32	Violet	94	KEGG:04640 Hematopoietic cell lineage (2.48E-06)	AFF3
33	Dark Olive Green	91	KEGG:hsa04662 B-cell receptor signaling pathway (5.0E-2)	LRP1
36	Yellow Green	87	KEGG:04611 Platelet activation (3.01E-07)	TREML1
44	Floral White	6699	KEGG:03013 Nucleocytoplasmic transport (3.78E-14)	METTL23
45	Dark Orange	120	KEGG:hsa03008 Ribosome biogenesis in eukaryotes (9.20E-6)	SNORD104
46	Brown	39	No significant KEGG pathway.	TSPY4

Note: Hub gene corresponds to the gene with the highest intra-module connectivity. Size refers to the number of genes in a module. Modules sky blue and brown have no significant KEGG pathway.
